# Electroencephalogram-Based Motor Imagery Signals Classification Using a Multi-Branch Convolutional Neural Network Model with Attention Blocks

**DOI:** 10.3390/bioengineering9070323

**Published:** 2022-07-18

**Authors:** Ghadir Ali Altuwaijri, Ghulam Muhammad

**Affiliations:** Department of Computer Engineering, College of Computer and Information Sciences, King Saud University, Riyadh 11543, Saudi Arabia; 438203980@student.ksu.edu.sa

**Keywords:** electroencephalogram, motor imagery, convolutional neural network, attention mechanism, brain–computer interface

## Abstract

Brain signals can be captured via electroencephalogram (EEG) and be used in various brain–computer interface (BCI) applications. Classifying motor imagery (MI) using EEG signals is one of the important applications that can help a stroke patient to rehabilitate or perform certain tasks. Dealing with EEG-MI signals is challenging because the signals are weak, may contain artefacts, are dependent on the patient’s mood and posture, and have low signal-to-noise ratio. This paper proposes a multi-branch convolutional neural network model called the Multi-Branch EEGNet with Convolutional Block Attention Module (MBEEGCBAM) using attention mechanism and fusion techniques to classify EEG-MI signals. The attention mechanism is applied both channel-wise and spatial-wise. The proposed model is a lightweight model that has fewer parameters and higher accuracy compared to other state-of-the-art models. The accuracy of the proposed model is 82.85% and 95.45% using the BCI-IV2a motor imagery dataset and the high gamma dataset, respectively. Additionally, when using the fusion approach (FMBEEGCBAM), it achieves 83.68% and 95.74% accuracy, respectively.

## 1. Introduction

Brain–computer interface (BCI) systems interact between humans and machines without physical contact. The recent progress in this area has enabled devices to be controlled by brain signals [1]. The most used brain signals are electroencephalography (EEG) signals since they are non-invasive (measured from the scalp), have a high time resolution, and are relatively inexpensive [2,3,4]. Dealing with EEG signals is challenging because the signals are weak, may contain artefacts, are dependent on the patient’s mood and posture, and have low signal-to-noise ratio [5].

To measure this signal, researchers use an elastic cap worn in the head where the EEG electrodes are fitted. Such arrangement ensures that each experiment session’s data are collected from the same area on the scalp [6]. An EEG signal is a combination of numerous frequencies of the brain signal. The majority of studies [7] employ a frequency range of 0–35 Hertz. However, we choose the whole band of frequencies without focusing on band-limited signals.

This paper focused on EEG signals based on motor imagery (MI), which is the act of envisioning limb movement. A subject’s MI data are generated when he or she imagines moving a particular limb. In the early 2000s, researchers discovered that using common spatial patterns (CSP) was the best technique to identify EEG-based MI (EEG-MI) signals. For this approach, a collection of linear transformations, also known as spatial filters or distance optimizers, is sought over a variety of classes. The energy of the filters constitutes the feature set, which is fed to a support vector machine (SVM) [8].

The decoding of EEG-MI is not only related to tremendous prospective but also crucial applications such as healing [9,10], gaming [11], and robotics [12,13]). However, with regard to data gathering and classification methodologies, there are significant limitations. The goal of this study is to develop a deep learning-based classification model, which can accurately decode EEG-MI signals with high kappa values, which is an evaluation statistic that relates between the system accuracy and coincidental accuracy. Even though deep learning has achieved success in various disciplines, its use to classify EEG-MI signals lacks good performance. This is partially owing to the weak signal-to-noise ratio, the presence of motion artefact and noise, and spatial correlation of the signals.

The fundamental challenge with EEG-MI categorization, according to preliminary observations, is that it is a more subject-specific task, which implies that every individual has distinctive characteristics that help the system classify the MI more effectively. The rehabilitation of stroke patients requires the use of a subject-specific EEG-MI classification scheme. This problem can be solved by multi-branch and multi-scale structures which make the model more generalized; however, these structures are often computationally expensive and hence, require much time to train. Therefore, we propose a lightweight deep learning-based EEG-MI model, which conforms the subject-specific task with fixed hyperparameters.

Contributions: The paper has the following main contributions:

- Develops a lightweight deep learning-based multi-branch model to classify EEG-MI signals.
- Applies attention mechanism to the proposed model to improve the accuracy.
- Develops a general model that can perform well with fixed hyperparameters.
- Investigates the effect of the fusion technique in the proposed model.
- Validates the efficiency and strength of the model in data variations by using multiple datasets.

The following is how the paper is organized. The literature review is provided in Section 2. The suggested Multi-Branch EEGNet with Convolutional Block Attention Module (MBEEGCBAM) is presented in Section 3. Section 4 provides experimental results and discussion, while Section 5 concludes the paper.

## 2. Background

### 2.1. Related Work

In the handcrafted method, the feature extraction and the classification are done separately [14,15], while in deep learning, these can be done in just one processing block. This gives it an advantage to success, especially in medical signals [16,17]. The most often utilized model in EEG-MI related tasks is convolutional neural networks (CNNs) [18,19,20,21,22], but deep belief networks (DBN) [19], stacked autoencoders (SAEs) [20], and recurrent neural networks (RNNs) [19,23] have also been utilized. In the processing of EEG-MI data, CNN offers several benefits, including the capacity to acquire time-based and spatial information concurrently, the facility to exploit the hierarchical structure of specific signals, and to provide excellent precision on large datasets.

CNN models are now applied in a variety of domains, including EEG-MI. The majority of articles that use deep learning to identify EEG-MI fall into one of four categories, depending on the input structure. Different features, spectral representation, raw signals, or topological maps can all be used as input formulations [7]. In determining the input formulation to employ, the design of the model is crucial.

Some researchers have done some preprocessing of EEG signals before feeding them into a CNN. Sakhavi et al. presented one such approach in [24]. In the EEG recordings, the authors applied the filter-bank CSP (FBCSP) [25], then retrieved temporal information and applied channel-wise convolution. The authors used the BCI-IV2a dataset to test their approach, which yielded an average accuracy of 74.46%. 

Inspired by the FBCSP, a ConvNet was proposed to classify EEG-MI signals; the input to the ConvNet is raw EEG data [26]. In [18], two models were presented; the first one was the ShallowConvNet, and the second one was the DeepConvNet. The ShallowConvNet has fewer layers while the DeepConvNet is a deeper version of the ShallowConvNet having extra aggregating layers. In [27], EEGNet was proposed as a dense form of prior techniques. It involves a depthwise convolution and a separable convolution, which permits the network for a reduction in the number of parameters. Riyad et al. [28] proposed a structure that has the EEGNet followed by an inception block. In [29], the authors proposed temporal convolutional networks (TCNs) with the EEGNet. All of these models address EEGNet’s flaws, which limiting network volume and leading to overfitting. Because of these flaws, even with a larger network, the throughput is still subpar. A multi-branch model, which absorbs attributes from different branches, is recommended as a consequence of this.

Amin et al. developed a multilayer fusion approach to EEG-MI classification in [30]. The features from different layers of a CNN are fused using several fusion strategies. They tested using two classification approaches: subject-specific and cross-subject, and the test included two datasets: BCI-IV2a and high gamma dataset (HGD). In both datasets, the multilayer CNNs with MLP (MCNN) model produced more accuracy than the other state-of-the-art (SOTA) models in the subject-specific classification. Furthermore, the multilayer CNNs with cross-encoding autoencoders (CCNN) model showed a significant accuracy gain in the cross-subject classification. The same researcher proposed in [31] a two-attention-block inception model. This produced decent accuracy in the BCI-IV2a dataset (74.7%) and HGD (94%).

Recently, a multi-branch 3D CNN to maintain spectro-temporal characteristics was proposed in [22]. The authors embodied the three dimensions as a series of two-dimensional representations based on the sensors’ locations and then used the temporal information as the third dimension. To increase the number of training samples, the authors utilized a cropped method. Their finding showed that the proposed 3D CNN outperformed the three single networks in terms of accuracy. In another work, 3D filters were used for the 3D CNN-based EEG-MI classification model [32]. In practice, the 3D filter is harder to construct, whereas the 1D filter is simpler. A network having three one-dimensional filters to cover all three dimensions in 3D may outperform traditional convolutional networks while requiring much less computation, according to researchers in [33]. In our proposed model, there is a 2D CNN with two 1D filters applied along time/space; this model can lessen computation while increasing the model’s skill to cope with subject-specific difficulties compared to 3D filters.

The authors in [34] introduced a CP-MixedNet structure, where each of the convolution layers collects EEG temporal information at different scales. Using the self-attention process, the authors of [35] developed a spatial-temporal representation of raw EEG data. When coding EEG-MI channels, the spatial self-attention module was used. The raw signal was filtered to various band ranges by the authors of [36] to produce three band-limited signals. Each band-limited signal is passed through three parallel branches with varied filter sizes. This caused a massive number of parameters more than 1215 K for the whole system. The system’s use in many applications is limited as a result of this scenario. Furthermore, because the filter size did not vary, the influence of changing localities in channels was not accounted for in the model. In [37], the authors proposed a more sophisticated approach based on a temporal-spectral-based squeeze-and-excitation (SE) feature fusion network (TS-SEFFNet). It is a computationally expensive network with a huge number of parameters.

A combination between the multi-scale and the attention was proposed in [38]. Based on the attention process, the authors developed a multi-scale convolutional neural network using attention mechanism for fusion (MS-AMF). In the BCI-IV2a dataset, the experimental findings demonstrated that the network had superior classification than the baseline technique with 79.9% average accuracy. However, this model has a preparation part for the data before inputting them into the model. Jia et al. [39] proposed a big model that has several branches on each different scale; this increased the computation complexity. It contains five parallel branches each having an inception block followed by a residual block and a SE. The EEG Inception block (EIB) has four parts: three 1D convolutions (with different kernel sizes that gradually increase among all EIBs) and pooling operations. The authors did the experiments on two public BCI competition datasets: BCI-IV2a and BCI-IV2b datasets achieving 81.4%, and 84.4% accuracies, respectively.

In our previous work [40], we examine the multi-branch CNN in classifying the raw EEG-MI signal with fewer parameters. In this study, we investigate the effect of adding attention blocks to the multi-branch EEGNet.

### 2.2. Datasets

In this research, we evaluated our proposed model with two frequently used public EEG-MI datasets. Data from nine people were gathered using 22 EEG electrodes in the BCI Competition IV-2a dataset (BCI-IV2a) at a rate of 250 Hz [41]. In addition, data on eye movement were collected using three additional electrooculography (EOG) channels. There are four MI classes: left hand, right hand, feet, and tongue.

To validate the proposed model’s robustness against data variations, we evaluated it using another dataset which is the HGD. The HGD has more trials than the BCI-IV2a, and has four classes: left hand, right hand, both feet, and rest. The HGD was collected in a controlled setting from 14 volunteers [18]. The data were collected using a total of 128 channels, only 44 related to MI, at a sampling frequency of 500 Hz.

## 3. Method

### 3.1. EEG Data

For the BCI-IV2a dataset, from the onset of the pre-cue through the completion of each trial, we obtained 4.5 s of data of sampling frequency 250 Hz (250 × 4.5 = 1125 samples). Each trial produced a data matrix of dimension (22 × 1125).

Downsampling the HGD dataset from 500 Hz to 250 Hz resulted in an improvement in the data quality. Additionally, channels were decreased from 128 to 44 to eliminate repetitive information. We excluded the electrodes not connecting to the motor imagery area. We picked only 44 sensors with C in their name (according to the database description) as they cover the motor cortex. To be consistent with the BCI-IV2a dataset, we used each trial of 4.5 s (0.5 s before the cue to the end of the trial) to produce 1125 samples per trial with a data matrix of dimension (44 × 1125) [35]. There were no further filters used, and each channel was uniform. The accuracy was calculated across trials for the same subject (within subject).

### 3.2. EEGNet Block

Local connection, invariance to location, and invariance to local changeover are three fundamental properties of the cerebral cortex. CNN’s primary concept is to use a filter to examine the influence of adjacent neurons [42,43]. The filter size we use is determined by the data type and the feature map we wish to create. The first block in our proposed model is the EEGNet which was introduced in [27]. The EEGNet block contains three convolution operations with varied window sizes, which are defined by the kernel size.

**Figure 1:**
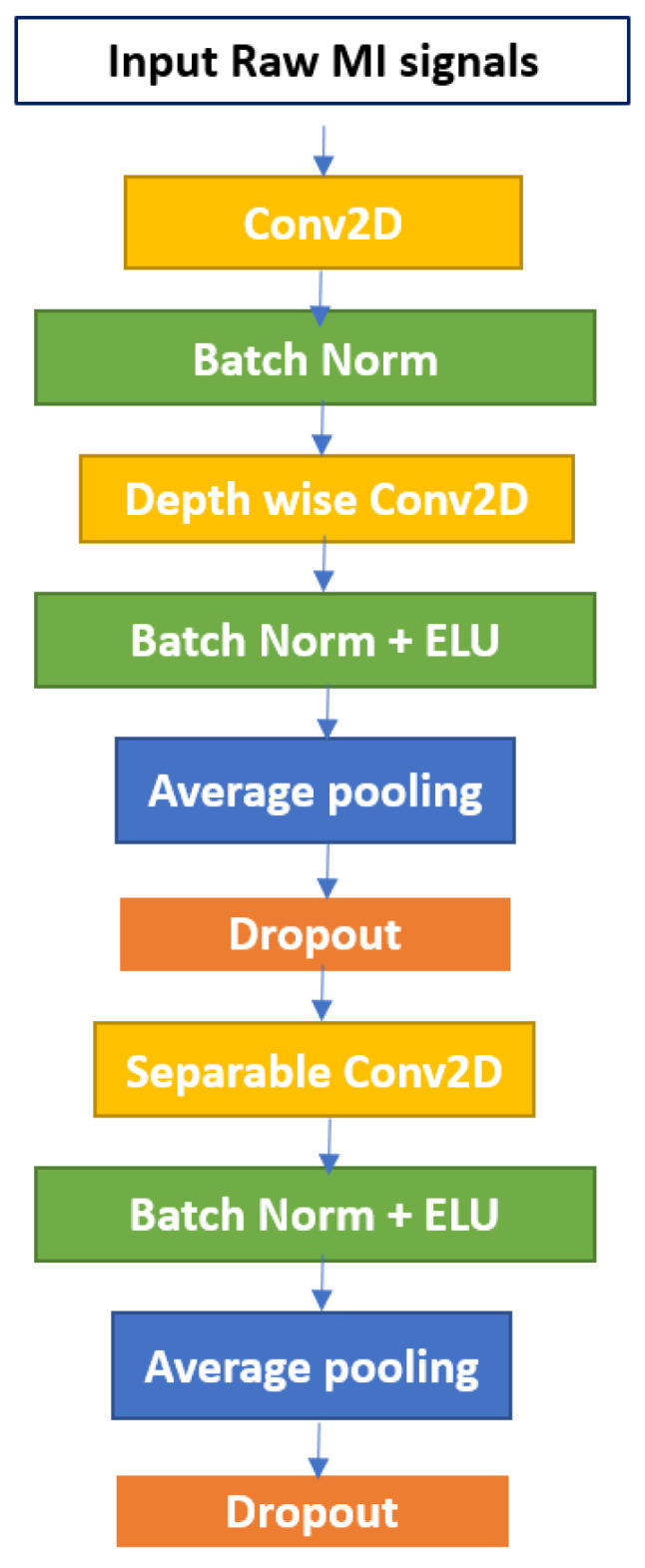
The EEGNet block.

The first convolution layer uses 2D filters followed by a batch normalization. Batch normalization aids in the acceleration of training and the regularization of the model [44]. The second convolutional layer uses depthwise convolution followed by batch normalization and activation function in the form of an exponential linear unit (ELU), average pooling, and dropout. The third convolutional layer uses separable convolution. A simplified architecture of the EEGNet is shown in Figure 1.

### 3.3. CBAM Attention Block

Attention is well established to play a significant influence in human perception. A human uses a sequence of limited sights and categorically focuses on significant sections of the image to apprehend the visual meaning [45]. From this idea comes the attention mechanism in deep learning. It is a module that can be added to the model to focus on relevant attributes and ignore others.

**Figure 2:**
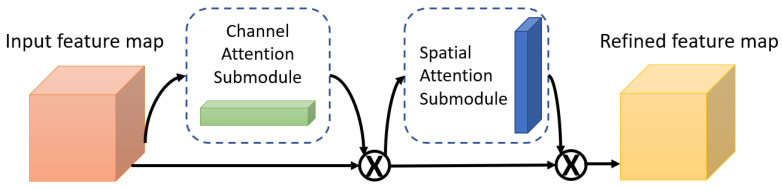
The Convolutional Block Attention Module (CBAM).

**Figure 3:**
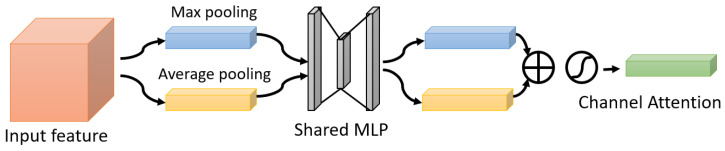
The Channel Attention Submodule.

**Figure 4:**
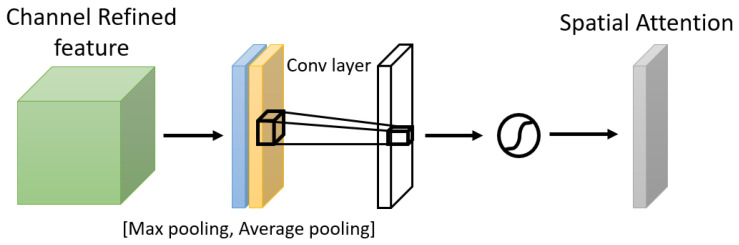
The Spatial Attention Submodule.

One of the attention modules is the Convolutional Block Attention Module (CBAM) described in [46], where the authors built a module to emphasize significant characteristics along the channel and spatial axes. Each branch may learn ‘what’ and ‘where’ to pay attention in the channel and spatial axes by using the sequence of attention modules (as shown in Figure 2). Because the module learns which information to highlight or hide, it efficiently helps the flow of information across the network. CBAM has two submodules: the channel attention submodule and the spatial attention submodule. In the channel attention submodule, the input features from the preceding block are concurrently transmitted to the average pooling and max-pooling layers. The features map generated by both pooling layers is then transmitted to a shared network, which is made up of an MLP with one hidden layer. In this hidden layer, a reduction ratio was used to reduce the number of activation maps which reduces the parameter overhead. After applying the shared network to each pooling feature map, element-wise summing is used to merge the output feature maps. Then, to generate the feature vectors that will be the input for the spatial attention submodule, the element-wise multiplication is used between the output feature map from the channel attention submodule and the input features map for the attention module. When calculating spatial attention, the channel axis average-pooling and max-pooling processes are used. As a result, a convolution layer is used to build an efficient feature descriptor. Both submodules are presented in Figure 3 and Figure 4.

### 3.4. Proposed Models

We propose a multi-branch EEG-MI classification system, where each branch has its own set of parameters to deal with the subject-specific problem. More specifically, we use three branches in the proposed system. Using the suggested technique, the convolution size, number of filters, dropout probability, and attention parameters may be determined for all subjects. It is also possible to tailor the model to a certain topic at the same time as increasing its applicability. In the first convolutional layer, based on local and global modulations, the model learns temporal properties and spatial attributes based on spatially distributed unmixing filters.

The proposed method Multi-Branch EEGNet with Convolutional Block Attention Module (MBEEGCBAM) can be divided into two parts: EEGNet block and Convolutional Block Attention Module (CBAM). Those basic blocks, EEGNet and CBAM, contain layers as described in [27,46], respectively.

**Figure 5:**
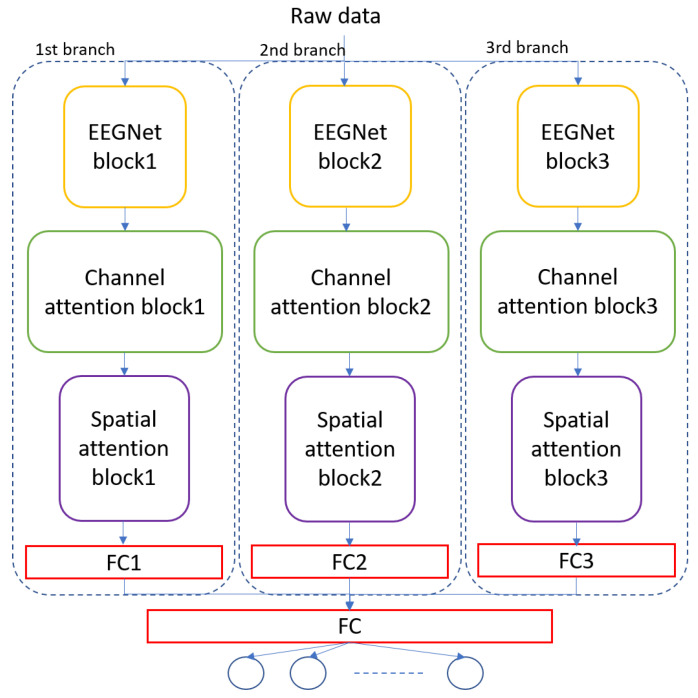
Architecture of the proposed model, MBEEGCBAM.

**Figure 6:**
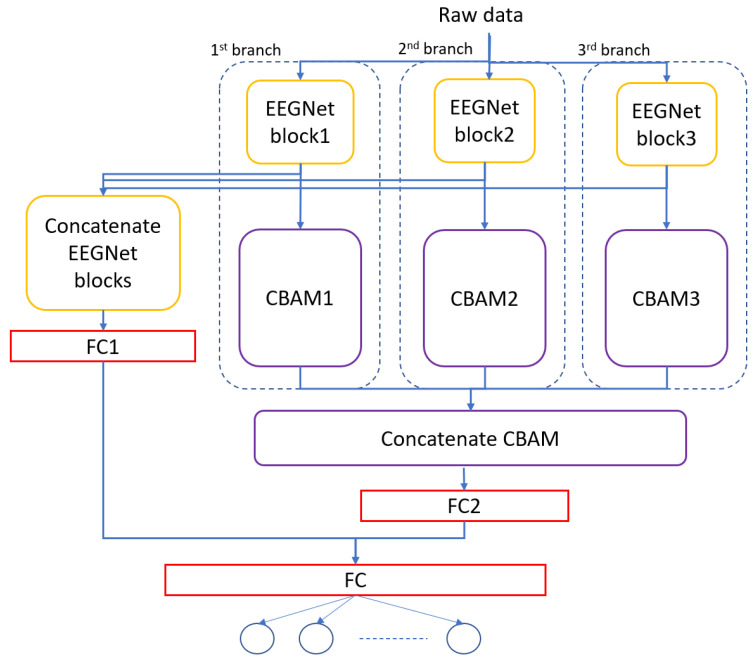
Architecture of the proposed model, FMBEEGCBAM.

The architecture of the MBEEGCBAM is shown in Figure 5. It has three different branches, each branch has an EEGNet block, channel attention block, and spatial attention block followed by a fully connected layer. Each branch has a varied number of parameters to capture different features. Moreover, after the improvement shown by the fusion in medical signals and images [47,48,49], we investigate the effect of fusion of the output feature maps from EEGNet blocks with the output from the EEG-CBAM blocks to reduce feature loss and construct a comprehensive feature map. For that, we propose the FMBEEGCBAM model (Figure 6) that has the same blocks and connections as in the MBEEGCBAM model with an extra step. In this model, we add two concatenate layers: one after the EEGNet blocks and the other one after the CBAM blocks, then we flat and fuse both concatenate layers before using the fused layer as input into the softmax layer for classification. We test our models in BCI-IV2a and HGD, which are two benchmark datasets in MI EEG classification.

### 3.5. Training Procedure

In the realm of EEG-MI research, the emotional and physical state of research volunteers can vary greatly. For that, we employed the within-subject approach to classifying the data in this research [30]. For both datasets, one session was used for training and the other was used for testing. Global hyperparameters, which were obtained in our previous work [40], were employed for all subjects, as shown in Table 1. The learning rate was 0.0009, batch size was 64, and the number of epochs was 1000. The Adam optimizer was used, and the cost function was the cross-entropy error function.

## 4. Experiments

The Tensorflow deep learning library with Keras API was used in all experiments in Google’s Colab environment.

### 4.1. Performance Metrics

To analyze our models, we used the following performance metrics: accuracy (%), precision, recall, F1 score, and Cohen’s Kappa test.

### 4.2. Overall Comparison

Table 2 shows the performance comparison between the proposed models and other SOTA models. In particular, the average classification accuracies, Kappa values, and F1 scores obtained by the FBCSP [25], ShallowConvNet [18], DeepConvNet [18], EEGNet [27], CP-MixedNet [34], TS-SEFFNet [37], MBEEGNet [40], and MBShallowCovNet [40] from the BCI-IV2a and HGD datasets are summarized in Table 2. Our methods have the highest average accuracy, Kappa, and F1 score as can be observed. Moreover, we compared our results with those of our previous work [40] which contains lightweight multi-branch models without attention blocks. We found that the attention block improves the accuracy by around 1%.

**Figure 7:**
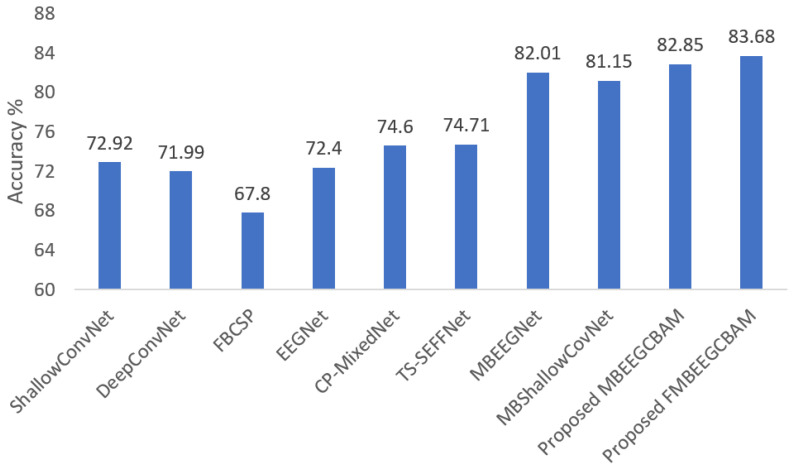
Average classification accuracy on the BCI-IV2a dataset. The methods compared are ShallowConvNet [18], DeepConvNet [18], FBCSP [25], EEGNet [27], CP-MixedNet [34], TS-SEFFNet [37], MBEEGNet [40], MBShallowConvNet [40], the proposed MBEEGCBAM, and the proposed FMBEEGCBAM.

**Figure 8:**
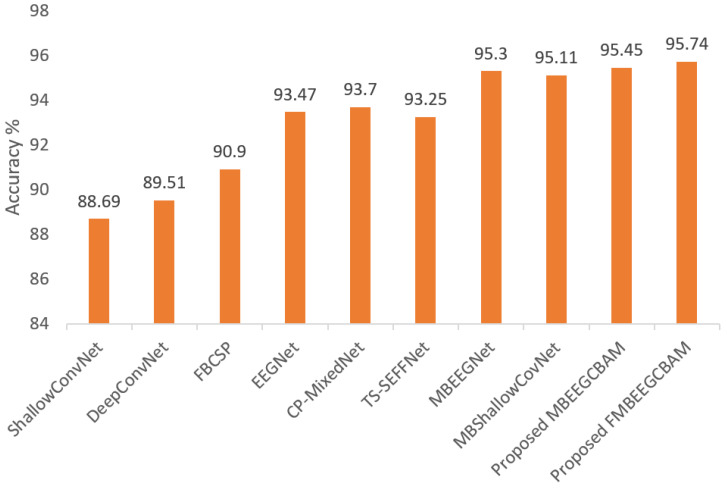
Average classification accuracy on the HGD. The methods compared are ShallowConvNet [18], DeepConvNet [18], FBCSP [25], EEGNet [27], CP-MixedNet [34], TS-SEFFNet [37], MBEEGNet [40], MBShallowConvNet [40], the proposed MBEEGCBAM, and the proposed FMBEEGCBAM.

Using the two public datasets, we evaluate the performance of our models. Figure 7 and Figure 8 show how our methods performed against the SOTA models in the BCI-IV2a and HGD. From the figures, we can see that the proposed models achieve at least 8.14% higher accuracy than other baseline models in the BCI-IV2a. However, in HGD the improvement was by around 2% in both MBEEGCBAM and FMBEEGCBAM.

### 4.3. Results of MBEEGCBAM

The proposed model was trained on session “T” from the BCI-IV2a dataset, while in the HGD, the proposed model was trained in all sessions in the dataset except the last two sessions which were kept for the testing. In the experiments, the within-subject or subject-specific approach was used.

**Figure 9:**
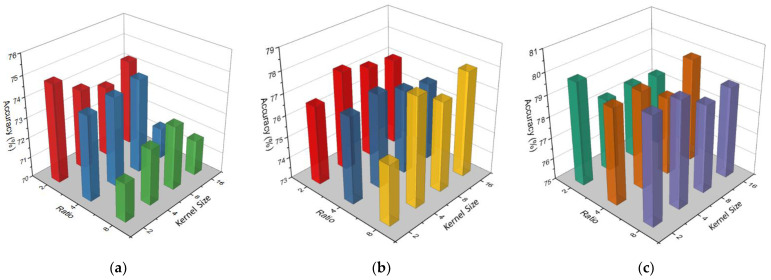
Accuracy comparison between different kernel sizes and ratio in CBAM block on EEGNet Block 1 (**a**), EEGNet Block 2 (**b**), and EEGNet Block 3 (**c**).

One of the main focuses of this study was to find the optimal hyperparameters that can advance the accuracy with less complication. Therefore, first, we found the best hyperparameters in the EEGNet block by performing multiple experiments. Then, we carried out other experiments to choose the best reduction ratio and kernel size in the CBAM block. Figure 9 shows the accuracy comparison between different kernel sizes and Ratios in CBAM blocks on different EEGNet blocks. As we can see from Figure 9, in EEGNet Block 1 (Figure 9a), the maximum accuracy was attained (74.83%) at ratio 2 and kernel size 2 × 2. Ratios 2 and 4 normally give better accuracy in EEGNet Block 1; however, ratio 8 gave better accuracy in EEGNet Block 2 and Block 3. On the hand, kernel size 8 × 8 and 4 × 4 gave better accuracy in EEGNet Block 1 and Block 2 while kernel size 2 × 2 provided the best accuracy in EEGNet Block 3. We chose Ratio 2 for EEGNet Block 1 and Ratio 8 for EEGNet Block 2 and Block 3; for the kernel size, we chose 2 × 2 for EEGNet Block 1 and Block 3 and size 4 × 4 for EEGNet Block 2. The hyperparameters that we used in the CBAM block in each branch of our proposed models are mentioned in Table 1.

Table 3 shows the performance of each branch separately, as well as the multi-branch model without attention blocks compared with our proposed model using the BCI-IV2a dataset. From the table, we can see that the proposed method, which is a combination of different EEGCBAM branches, enhances the performance of EEG-MI classification.

The comprehensive findings of MBEEGCBAM using the BCI-IV2a dataset and HGD are shown in Table 4 and Table 5. In the tables, LH, RH, F, and Tou represent left hand, right hand, feet, and tongue MI classes, respectively. Using Wilcoxon signed-rank test, there is a significant increase with *p* < 0.05 in the average accuracy and Kappa value using MBEEGCBAM compared to other SOTA models described in [18,29,34,37].

### 4.4. Results of FMBEEGCBAM

To study the effect of the fusion of multi-branches, we added a connection between the output feature maps from EEGNet blocks with the output from the EEG-CBAM blocks. Table 6 and Table 7 show the detailed result using both datasets, the BCI-IV2a and the HGD. From the tables, we can see that the proposed fusion model improves the classification accuracy in six subjects out of nine in the BCI-IV2a dataset, while in the HGD, eight subjects have an improvement in the accuracy. The drawback of this model is the increase in the number of parameters. The fusion model has 3808 parameters more than the MBEEGCBAM model with around a 1% increase in the classification accuracy.

### 4.5. Feature Discrimination Discussion

**Figure 10:**
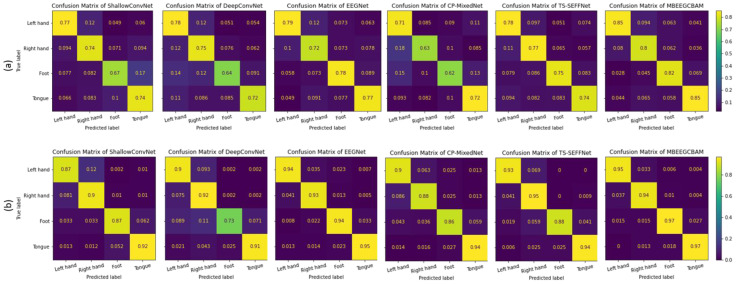
The average confusion matrices for all subjects on both datasets (**a**) the BCI-IV2a, (**b**) the HGD.

**Figure 11:**
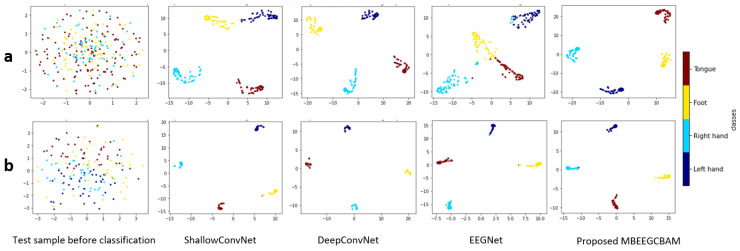
The t-SNE visualization of test sample features before and after classification using the comparative methods in the third subject in (**a**) the BCI-IV2a, and (**b**) the HGD.

Using a confusion matrix, we demonstrate the competence of features obtained by the proposed MBEEGCBAM for different MI classes. Figure 10 shows the confusion matrixes of the proposed model and the SOTA models in both datasets. We see that the proposed MBEEGCBAM significantly improved accuracy in four MI tasks across both datasets, especially in the “Foot” task, which reached an average increase of 12.8% in the BCI-IV2a and 11.4% in the HGD. The rest of the tasks increased by around 9.13% in the BCI-IV2a and 3.5% in the HGD. To study the discriminative nature of the features obtained by the MBEEGCBAM, the t-SNE was used to visualize the features [50] (see Figure 11). Compared to ShallowConvNet [18], DeepConvNet [18], and EEGNet [27], the proposed MBEEGCBAM model extracted more separable features from EEG-MI.

## 5. Conclusions

In this paper, we propose lightweight multi-branch models with attention, which improve the performance of EEG-MI classification with fewer parameters. The multi-branch model concatenates different features from three branches. When compared to other SOTA models, our model exhibits promising results in terms of accuracy, Kappa value, and F1 score. Our results were more accurate than other multi-branch models and required less human intervention. The study used the BCI-IV2a dataset and the HGD dataset, both of which are freely available. The experiment used a within-subject method, with global hyper-parameters applied to all subjects in both datasets. The proposed MBEEGCBAM had an average classification accuracy of 82.85% on the BCI-IV2a dataset, while that of the proposed FMBEEGCBAM was 83.68%. The average accuracy on the HGD in MBEEGCBAM and FMBEEGCBAM was 95.45% and 95.64%, respectively. In the future, we want to apply different fusion strategies in the proposed models.

## Figures and Tables

**Table 1:** Global hyper-parameters used in proposed models.

Branch	Block	Activation Function	Hyperparameter	Value
First branch	EEGNet Block	elu	Number of temporal filters	4
			Kernel size	16
			Dropout rate	0
	Attention Block	relu	Ratio	2
			Kernel size	2
Second branch	EEGNet Block	elu	Number of temporal filters	8
			Kernel size	32
			Dropout rate	0.1
	Attention Block	relu	Ratio	8
			Kernel size	4
Third branch	EEGNet Block	elu	Number of temporal filters	16
			Kernel size	64
			Dropout rate	0.2
	Attention Block	relu	Ratio	8
			Kernel size	2

**Table 2:** The comparison summary of classification performance in proposed models.

Datasets	Methods	Accuracy (%)	Kappa	F1 Score
BCI-IV2a	FBCSP [25]	67.80	Not Available (NA)	0.675
	ShallowConvNet [18]	72.92	0.639	0.728
	DeepConvNet [18]	71.99	0.627	0.719
	EEGNet [27]	72.40	0.630	-
	CP-MixedNet [34]	74.60	NA	0.743
	TS-SEFFNet [37]	74.71	0.663	0.757
	MBEEGNet [40]	82.01	0.760	0.822
	MBShallowCovNet [40]	81.15	0.749	0.814
	MBEEGCBAM (proposed)	82.85	0.771	0.830
	FMBEEGCBAM (proposed)	83.68	0.782	0.838
HGD	FBCSP [25]	90.90	NA	0.914
	ShallowConvNet [18]	88.69	0.849	0.887
	DeepConvNet [18]	89.51	0.860	0.893
	EEGNet [27]	93.47	0.921	0.935
	CP-MixedNet [34]	93.70	NA	0.937
	TS-SEFFNet [37]	93.25	0.910	0.901
	MBEEGNet [40]	95.30	0.937	0.954
	MBShallowCovNet [40]	95.11	0.935	0.951
	MBEEGCBAM (proposed)	95.45	0.939	0.955
	FMBEEGCBAM (proposed)	95.74	0.943	0.958

**Table 3:** The classification performance in different models.

Methods	Accuracy (%)
EEGCBAM1	74.83
EEGCBAM2	78.02
EEGCBAM3	79.92
MBEEGNet [40]	82.01
MBEEGCBAM (proposed)	82.85

**Table 4:** Performance metrics on the BCI-IV2a dataset using the MBEEGCBAM.

		1	2	3	4	5	6	7	8	9	Avg.	Std. Div.
Accuracy (%)		91.09	65.87	94.52	77.88	81.87	64.48	93.38	89.84	86.69	82.85	0.113
K value		0.881	0.545	0.927	0.705	0.758	0.526	0.912	0.865	0.822	0.771	0.151
F1 score		0.912	0.662	0.945	0.782	0.821	0.644	0.937	0.899	0.869	0.830	0.113
Precision	LH	0.941	0.601	0.968	0.84	0.919	0.562	0.982	0.914	0.904	0.848	0.157
	RH	0.943	0.539	0.958	0.795	0.777	0.584	0.84	0.899	0.827	0.796	0.147
	F	0.936	0.764	0.915	0.634	0.724	0.714	0.958	0.847	0.862	0.817	0.113
	Tou.	0.824	0.729	0.94	0.848	0.854	0.719	0.955	0.935	0.876	0.853	0.086
	Avg.	0.911	0.658	0.945	0.779	0.819	0.645	0.934	0.899	0.867	0.828	0.114
Recall	LH	0.909	0.580	0.932	0.762	0.842	0.606	0.838	0.956	0.854	0.809	0.135
	RH	0.94	0.535	0.986	0.716	0.909	0.630	0.979	0.913	0.785	0.821	0.163
	F	0.870	0.882	0.937	0.856	0.795	0.636	0.970	0.875	0.876	0.855	0.096
	Tou.	0.929	0.669	0.928	0.807	0.748	0.703	0.972	0.856	0.967	0.842	0.116
	Avg.	0.912	0.667	0.946	0.785	0.823	0.644	0.940	0.90	0.870	0.832	0.113

**Table 5:** Performance metrics on the HGD dataset using the MBEEGCBAM.

Subject/Metric	Accuracy (%)	K Value	Precision	Recall	F1 Score
S1	96.43	0.952	0.964	0.965	0.965
S2	93.52	0.914	0.935	0.939	0.937
S3	100	1	1	1	1
S4	96.90	0.959	0.969	0.969	0.969
S5	97.52	0.967	0.975	0.975	0.975
S6	98.80	0.984	0.988	0.988	0.988
S7	95.25	0.937	0.953	0.955	0.954
S8	96.90	0.959	0.969	0.969	0.969
S9	98.20	0.976	0.982	0.982	0.982
S10	89.93	0.866	0.899	0.903	0.901
S11	90.50	0.873	0.905	0.907	0.906
S12	96.35	0.951	0.963	0.964	0.964
S13	96.90	0.959	0.969	0.969	0.969
S14	89.09	0.855	0.891	0.895	0.893
Average	95.45	0.939	0.954	0.956	0.955
Std. Div.	0.034	0.045	0.034	0.033	0.033

**Table 6:** Performance metrics on the BCI-IV2a dataset using the FMBEEGCBAM.

		1	2	3	4	5	6	7	8	9	Avg.	Std. Div.
Accuracy (%)		92.96	68.33	96.75	80.39	79.78	69.73	91.18	88.77	85.21	83.68	0.099
K value		0.906	0.578	0.957	0.739	0.730	0.596	0.882	0.850	0.803	0.782	0.133
F1 score		0.931	0.684	0.968	0.806	0.800	0.699	0.915	0.889	0.853	0.838	0.099
Precision	LH	0.957	0.639	0.984	0.915	0.800	0.618	0.982	0.902	0.918	0.918	0.141
	RH	0.971	0.555	0.945	0.691	0.813	0.643	0.870	0.923	0.772	0.772	0.145
	F	0.965	0.831	0.970	0.741	0.814	0.789	0.955	0.822	0.857	0.857	0.083
	Tou.	0.825	0.709	0.971	0.870	0.764	0.740	0.840	0.904	0.861	0.861	0.083
	Avg.	0.929	0.684	0.968	0.804	0.798	0.697	0.912	0.888	0.852	0.852	0.099
Recall	LH	0.948	0.599	0.946	0.777	0.848	0.732	0.833	0.971	0.865	0.835	0.119
	RH	0.971	0.588	1.000	0.726	0.868	0.682	0.980	0.888	0.758	0.829	0.147
	F	0.854	0.854	0.958	0.912	0.711	0.656	0.912	0.859	0.848	0.840	0.097
	Tou.	0.959	0.697	0.969	0.820	0.784	0.732	0.949	0.841	0.947	0.855	0.105
	Avg.	0.933	0.685	0.968	0.809	0.803	0.700	0.918	0.890	0.854	0.840	0.099

**Table 7:** Performance metrics on the HGD dataset using the FMBEEGCBAM.

Subject/Metric	Accuracy (%)	K Value	Precision	Recall	F1 Score
S1	97.55	0.967	0.976	0.976	0.976
S2	96.29	0951	0.963	0.963	0.963
S3	100	1	1	1	1
S4	98.80	0.984	0.988	0.988	0.988
S5	98.20	0.976	0.982	0.982	0.982
S6	98.77	0.984	0.988	0.988	0.988
S7	94.43	0.926	0.944	0.944	0.944
S8	96.52	0.954	0.965	0.968	0.966
S9	98.77	0.984	0.988	0.988	0.988
S10	91.18	0.882	0.912	0.915	0.913
S11	88.82	0.851	0.888	0.890	0.889
S12	96.94	0.959	0.969	0.970	0.970
S13	96.52	0.954	0.965	0.968	0.966
S14	87.49	0.833	0.875	0.880	0.878
Average	95.74	0.943	0.957	0.959	0.958
Std. Div.	0.039	0.052	0.039	0.038	0.038

## Data Availability

Not applicable.
